# Towards using 3D cellular cultures to model the activation and diverse functions of macrophages

**DOI:** 10.1042/BST20221008

**Published:** 2023-02-06

**Authors:** Sean Cutter, Mark D. Wright, Nicholas P. Reynolds, Katrina Jean Binger

**Affiliations:** 1 Department of Biochemistry and Chemistry, La Trobe Institute for Molecular Science, La Trobe University, Bundoora, Victoria 3086, Australia; 2 Department of Immunology and Pathology, Alfred Medical Research and Education Precinct, Central Clinical School, Monash University, Melbourne, Victoria 3004, Australia; 3 Department of Biochemistry and Pharmacology, Bio21 Molecular Science and Biotechnology Institute, The University of Melbourne, Parkville, Victoria 3010, Australia

**Keywords:** 3D culture, bioengineering, bioprinting, cell cultures, extracellular matrix, macrophages

## Abstract

The advent of 3D cell culture technology promises to enhance understanding of cell biology within tissue microenvironments. Whilst traditional cell culturing methods have been a reliable tool for decades, they inadequately portray the complex environments in which cells inhabit *in vivo*. The need for better disease models has pushed the development of effective 3D cell models, providing more accurate drug screening assays. There has been great progress in developing 3D tissue models in fields such as cancer research and regenerative medicine, driven by desires to recreate the tumour microenvironment for the discovery of new chemotherapies, or development of artificial tissues or scaffolds for transplantation. Immunology is one field that lacks optimised 3D models and the biology of tissue resident immune cells such as macrophages has yet to be fully explored. This review aims to highlight the benefits of 3D cell culturing for greater understanding of macrophage biology. We review current knowledge of macrophage interactions with their tissue microenvironment and highlight the potential of 3D macrophage models in the development of more effective treatments for disease.

## Introduction

Traditional 2D cell culture on stiff, flat polystyrene substrates has provided a broad depth of knowledge on cellular processes, thereby increasing understanding of the human body and biology. However, the emergence of technologies such as bioreactors and bioprinting now permits the addition of a third dimension to these traditional culture methods. So far, three-dimensional (3D) cultures have been highly utilised in cancer research to better understand the contribution of tissue environments to the mechanisms of tumorigenesis, thereby better replicating *in vivo* conditions to improve outcomes in high-throughput drug screening assays. However, research fields such as immunology have yet to fully utilise this technique. Immune cells occupy diverse 3D spaces *in vivo* which can further change upon the infiltration of pathogens, inflammation, and tissue remodelling. Among these immune cells, macrophages are present in all tissues and exhibit a vast range of functions important for tissue homeostasis and host immunity. In this review, we discuss the environmental factors that are important for macrophage function and phenotypes, and how this information could be considered in the design of 3D systems for better understanding of macrophage biology *in vitro*. We also review recent studies which have utilised 3D cell cultures to investigate aspects of macrophage biology.

## Macrophages

Macrophages are innate immune cells that are known for their ability to ingest particles in a process known as phagocytosis. However, in the more than 100 years since their discovery, these cells are now appreciated to be more than just eponymous ‘big eaters' [1]. Macrophages are present within all human tissues and have a plethora of phenotypes ranging from the phagocytosis and elimination of invading pathogens to other processes which regulate essential homeostatic organ functions. One driver of macrophage phenotype is their developmental origin. During early embryonic development, waves of macrophage precursors populate developing tissues and are then retained within specific niches where they develop into macrophages focused on tissue-specific homeostatic operations [2]. These tissue resident macrophages (TRMs) have a capacity for self-renewal and persist within organs throughout the life of the organism. The roles which specific TRMs play to maintain tissue homeostasis varies greatly depending on their respective tissues. For example, brain TRMs known as microglia have roles in synaptic pruning, a process involving the clearance of excess synaptic connections [3]. In the lung, TRMs known as alveolar macrophages are responsible for surfactant clearance [4]. Osteoclasts are TRMs of the bone maintain bone density homeostasis, a process which can lead to osteoporosis if disturbed [5]. These diverse, tissue-specific, tasks imply that signals from the local tissue environment play a role in shaping TRM function.

In contrast, macrophages can be generated on command throughout adulthood from circulating hematopoietic precursors. This is the more recognised origin of macrophages whereby hematopoietic stem cells (HSCs) in the bone marrow develop into myeloid precursors and then Ly6C^+^ monocytes, which then travel throughout the circulation until an injurious or inflammatory signal is detected from a tissue. In the presence of the cytokine colony-stimulating factor 1 (CSF1), monocytes then infiltrate the tissue and differentiate into macrophages that can further respond to the threat at hand [6]. It is these tissue-infiltrating macrophages that are best recognised for protecting the host from pathogens and responding to organ damage. Because of their different ontogeny, TRMs and monocyte-derived macrophages are genetically distinct and thereby largely considered to be discrete cell types that cannot be recompensed by the other [7,8].

## 3D cell culture models

There is still a lot that we don't understand about macrophage responses within tissues. There are competing hypotheses that homeostatic TRM function is genetically instructed or steered by tissue microenvironments, and that their function can or cannot be recompensed by infiltrating monocyte-derived macrophages [7–9]. Likewise, identifying new molecules that attenuate or enhance monocyte-derived macrophage responses within tissues is an area of significant research due to the importance of macrophage function within various diseases. 3D cell culture is a rapidly emerging technology as more studies report these systems to be closer to *in vivo* tissues and promote natural cellular responses. Such technologies may therefore be beneficial to enhance understanding of macrophage biology. However, despite the appeal of 3D cell culture in providing a more physiological microenvironment, it comes with its own set of challenges including reproducibility, the control of mechanical properties like elasticity, porosity, and the unequal distribution of oxygen, nutrients, and waste products [10,11]. Several 3D culture methods have been developed, each with their own advantages and disadvantages (Table 1). These models differ in their physical and chemical characteristics and should be utilised depending on the requirements of the cell or system to be studied [12]. Some of the more commonly used models include the generation of cell aggregates or spheroids [13], and resuspension of cells within natural or synthetic bioscaffolds [14], which are enhanced with 3D bioprinting [15]. We now briefly review these various systems and their applicability for macrophage culture.

**Table 1:** Comparison of 3D culture methods

	Advantages	Disadvantages	Ref.
Hanging drop			
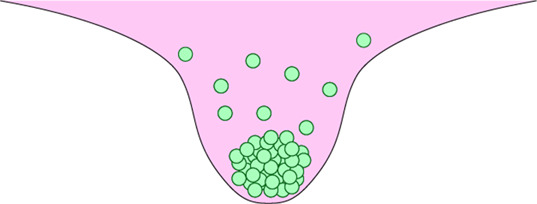	- Good supply of oxygen to cell aggregates - Flexible size dependent on cell concentration - Amenable for multicellular culture - Can be implanted into hydrogels	- Prolonged experiments require constant transfer and nutrient replenishment - Difficult to standardise - No cell–ECM interactions	[113,114]
Ultra-low attachment plates & magnetic levitation			
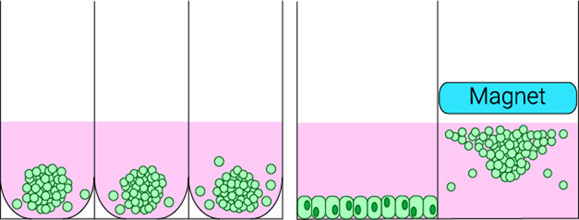	- Higher throughput and capacity for larger volumes - Amenable for multicellular culture - Can be implanted into hydrogels	- Unsuitable for adhesion-related studies - Difficult to standardise - No cell–ECM interactions	[18,115,116]
Bioreactor culturing			
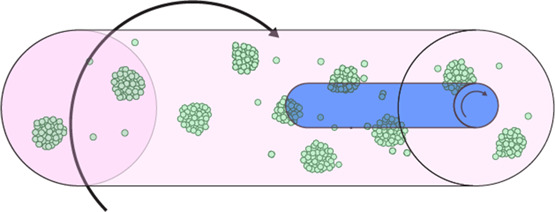	- Capacity for high volumes and aggregates - Highly controlled environment (temp, pH, oxygen/CO_2_…etc) - Can be combined with microcarrier technology to form consistent spheroids	- Cost ineffective; requires specialised equipment - Low throughput analysis - Difficulty standardising, particularly co-culture aggregates	[20,117,118]
Microcarriers			
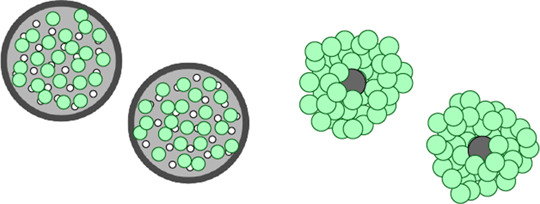	- Customizable bead sizes - Advantages as per other spheroid methodologies	- Disadvantages as per other spheroid methodologies	[21,119]
Bioscaffolds: natural, synthetic or composite			
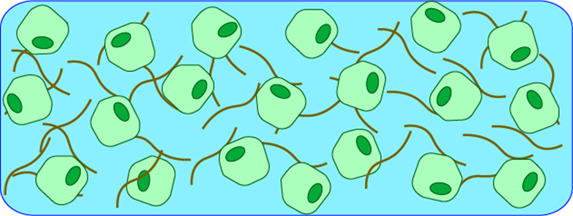	- Modelling of cell–ECM interactions - Ability to control cell adhesion - Easily adjustable biophysical parameters including stiffness and porosity - Can be enhanced with 3D bioprinting	- Difficulty producing cell aggregates/spheroids - Lower throughput - Reproducibility issues due to batch-dependent variability particularly with natural ECMs - Difficulty extracting cells for further analysis - Imaging challenges	[27,28,120–123]
3D bioprinting			
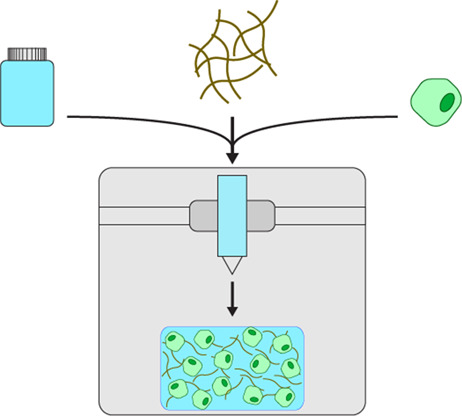	- Highly consistent and reproducible compared with other 3D culture methods - High throughput - Specific architecture and geometry of bioscaffolds can be designed - Other advantages as per bioscaffolds method	- Microtissues can be hard to formulate - Potential for poor cell seeding	[23,31,33,124]

### Cellular aggregates

The formation of cellular aggregates or ‘spheroids’ can be considered the simplest 3D culture model, simply requiring the self-aggregation of cells. These can be produced via a number of methods. In hanging-drop cultures, simple gravity causes cell–cell association and the formation of small aggregates. Microcarriers are comprised of beads coated with adhesive extracellular matrix (ECM) proteins like collagen that cells attach to [16,17]. Ultra-low attachment plates prevent cell adhesion to the plate surface, often promoted by further treating plates with a hydrophilic coating [18]. As a result, cells adhere to each other resulting in the formation of small cellular aggregates. Cells can also be treated with magnetic nanoparticles and subjected to a magnetic field in order to promote aggregation [19]. 3D spheroids can also be generated via constant agitation, such as in a rotating wall vessel [20], which not only promotes aggregation, but also maintains cell growth and differentiation [21]. Whilst ideal for high throughput cell–cell interaction studies, these methods are difficult to standardise and lack important cell–ECM interactions which are particularly critical for macrophage function (discussed later).

### Bioscaffolds

Bioscaffolds are one of the most common forms of 3D cell cultures. These models involve the encapsulation of cells within hydrogels comprised of synthetic (e.g. polyacrylamide or polyethylene glycol (PEG)) or natural polymers (e.g. alginate, collagen, or other ECM proteins). Nanofibrillar protein assemblies from ECM proteins such as collagen are frequently employed as natural bioscaffolds [22,23]. In these systems, cells are encapsulated within collagen fibres forming cell:scaffold interactions in three dimensions, akin to their natural tissue environment [24]. Cells can be mixed with bioscaffold material and gelation triggered by changes in pH or temperature. Additional extracellular matrix proteins can be readily incorporated either before or after gelation. At low concentrations, these structures are highly porous and readily allow the diffusion of media, supplements, gases, and small molecules [25]. However, in stiffer gels with increased fibrillar concentrations this porosity can be significantly reduced leading to limited nutrient exchange, removal of waste products and eventually cell death [26]. Detailed analysis of 3D suspended cells is a significant challenge with bioscaffolds models, often requiring further processing prior to analysis for example, degradation of hydrogels to produce single cell suspensions for flow cytometry or mass spectrometry analysis. Furthermore, the use of biological materials for hydrogel culture can be limited by batch variation and poor reproducibility.

Synthetic hydrocarbon-based polymers can also be used to form hydrogels for 3D cell culture and are often easier to manipulate. Using materials such as PEG it is possible to design hydrogels with specific architectures and flexibility by adjusting molecular density [27]. Additionally, through crosslinking of chemical groups structural features of the 3D network can be modified. For example, by varying the crosslinker structure in PEG-diester-dithiol gels, Jain et al. [28] were able to control gelation and degradation rates of their 3D matrices. Compared with biological hydrogels, synthetic scaffolds may offer improved reproducibility, however they often are bad mimics of ECM chemistry and architecture requiring further modification in order to be suitable for cell culture, including pH adjustment and ECM incorporation for cell adhesion. Whilst synthetic scaffolds have low biological mimicry of *in vivo* environments, they do pose several advantages in that they can be relatively easily tuned to a desired stiffnesses, have controllable degradation rates, and can be functionalised with drugs or proteins. These features make synthetic scaffolds ideal materials to be used in regenerative medicine where the aim is to implant sacrificial scaffolds into recovering tissue that boost regeneration [29,30]. The development of synthetic bioscaffolds may be particularly beneficial in the context of macrophages, which can acquire phenotypes that naturally promote tissue repair and are therefore attractive targets.

### Bioprinting

Bioprinting 3D scaffolds is a rapidly emerging system which has applications for tissue engineering and regenerative medicine [31]. Bioprinting utilises 3D printing technology, however rather than synthetic plastics, prints ‘bioinks' for cell culture. Bioinks range from natural materials such as collagen to commercially available mixtures suitable for 3D cell culture. The inks are usually mixed with cells prior to printing and need to be printable in their un-crosslinked state, meaning that they have sufficiently low viscosity to be extruded through a printer head. Immediately after printing the inks are chemically or photo-crosslinked to produce stiffer 3D hydrogels that retain their printed dimensions. Not only is this technique used to print biocompatible 3D cell scaffolds, but also complex tissues and organs [32]. There are several advantages to this technique, including the ability to print highly detailed structures with extreme precision that make it ideal for regenerative treatments [33]. Elsewhere, 3D bioprinting has been utilised to optimise tissue-mimetic models to investigate tumorigenesis and infectious diseases [23,34].

### Organs-on-a-Chip

Organs-on-a-Chip are state-of-the-art 3D systems which aim to model whole organs *in vitro* [35]. These models employ microfluidics devices to recapitulate parts of human organs including immunogenic sites such as lymph nodes and bone marrow. There are many varieties of these systems, but generally, cells are suspended within 3D hydrogels within microchannels that are then perfused with media. Chips can be made more advanced by the addition of endothelial cell-lined vascular channels [36] or co-culturing different cell types in multiple microchambers [37]. Immune-System-on-a-Chips are simply variations which incorporate immune cells. They have been used to study the migration of immune cells during inflammatory disease which was proposed to better replicate *in vivo* complexity compared with traditional transwell assays [38]. To date, there has been limited investigation of the effect of macrophages in these ‘chip’ models. Given their high abundance in tissues, the inclusion of macrophages would provide invaluable understanding of their role in disease and the maintenance of tissue homeostasis.

## Macrophage environmental stimuli

**Figure 1.**
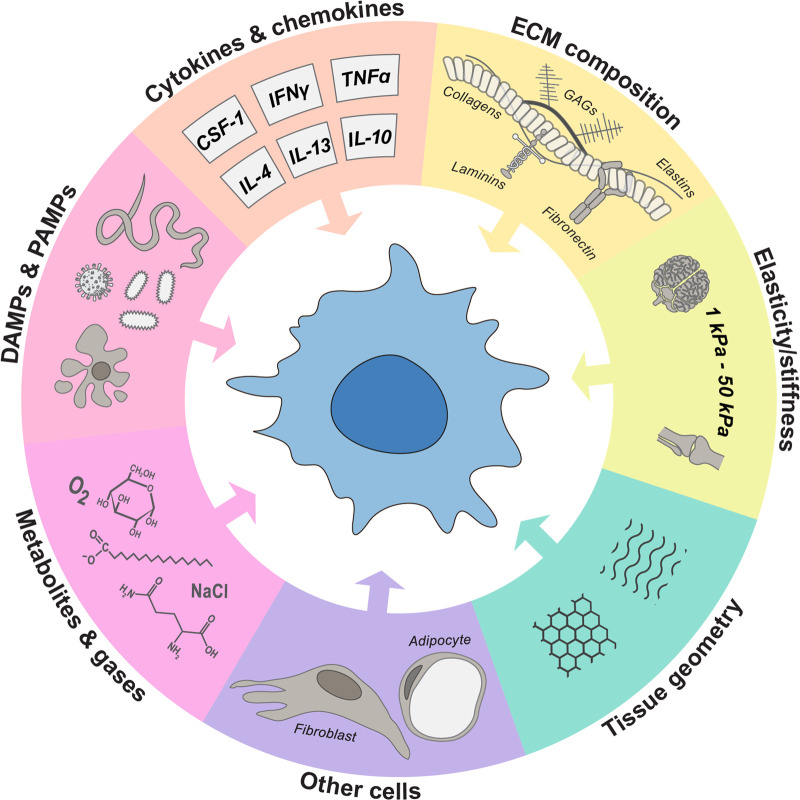
Environmental influences on macrophage biology. Tissue resident macrophages (TRM) respond to a variety of extracellular cues, all of which have been shown to influence their activation and function. Environmental factors that affect macrophage biology include: the extracellular matrix (ECM) composition, such as the concentration of collagen, elastin, laminin and fibronectin as well as glycosaminoglycans (GAGs); tissue stiffness and geometry; cytokine and chemokine signalling; damage-associated molecular patterns (DAMPs) and pathogen-associated molecular patterns (PAMPs); metabolite and dissolved gasses; and interactions with other cell types. Macrophage differentiation and survival are regulated by cytokines such as colony stimulating factor-1 (CSF-1), and are activated towards pro-inflammatory ‘M1' phenotypes in response to interferon γ (IFNγ) and tumour necrosis factor alpha (TNFα). Contrastingly, anti-inflammatory ‘M2' macrophages result from interleukin (IL)-4, IL-13 and IL-10 signalling. Altogether, these factors form a niche which determine TRM identity and ensure proper function, or promote activation to fight infection and restore homeostasis.

There are numerous biochemical and biophysical parameters which influence macrophage phenotype and function particularly immunogenic pathogens and cytokines. However, there is emerging evidence of additional tissue-specific properties that modulate macrophage phenotype, including the extracellular matrix (ECM) composition of tissues and their ligation of corresponding macrophage integrin receptors and other proteins, mechanical properties of tissues such as stiffness, the presence of other cells, and the concentration of small molecules (Figure 1). All these parameters may therefore be important to consider or incorporate when designing effective 3D models to model macrophage biology *in vitro*. Here we provide a general overview of how some of these factors influence macrophage function. The contribution of metabolites and other small molecules is not discussed here and we instead direct interested readers to reviews by [39,40].

### Infection and cytokine signalling

Macrophages are well known for their ability to sense and phagocytose invading pathogens. Pathogen sensing occurs via the detection of pathogen associated molecular patterns (PAMPs) via surface expressed pattern recognition receptors (PRRs); one example being lipopolysaccharides (LPS) found on the outer membrane of gram-negative bacteria which is recognised via toll-like receptor (TLR) 4 [41]. Detecting such a signal then results in the macrophage acquiring a so-called ‘M1' pro-inflammatory phenotype that aims to either directly kill any ingested pathogens via the production of enzyme nitric oxide synthase (iNOS) and antimicrobial nitric oxide (•NO) or attracting other immune cells by the production of pro-inflammatory cytokines such as interleukin (IL)-1β and tumour necrosis factor alpha (TNF)-α [42]. Similar mechanisms exist from macrophage sensing of endogenous danger signals damage associated molecular patterns (DAMPs), which are produced from dead or dying cells, subsequently stimulating PRRs and resulting in macrophage pro-inflammatory activation. There is an enormous body of literature describing the activation of macrophages via PAMPs, DAMPs and PRRs (for example [43]) and is not the focus of this review.

Cytokines are additional environmental signals that can potentiate macrophage activation in the presence of PAMPs/DAMPs, or that can directly induce macrophage phenotypes. During gram-negative bacterial infection where TLR signalling is activated, the production of interferon gamma (IFN-γ) by Th1 cells primes and potentiates macrophage activation and increases microbicidal functions. In contrast, upon infection with parasites such as helminths, a strong T helper 2 response is induced resulting in the production of interleukin (IL)-4, IL-13 and IL-10 [44]. Macrophages react to these cytokines through cytokine receptors which induce activation into so-called ‘M2' or anti-inflammatory phenotypes that promote parasite clearance and tissue repair [45]. For the latter, anti-inflammatory activated macrophages promote extracellular matrix (ECM) remodelling and repair by synthesising polyamines and substrates such as proline for the synthesis of ECM proteins [46]. Macrophage acquisition of anti-inflammatory phenotypes can also occur in the absence of helminths via the production of IL-4 and IL-13 by other mechanisms. For example, Qiu et al. [47] showed that when mice were exposed to environmental cold, IL-4-producing eosinophils migrate into white adipose tissue where they induce the infiltration and activation of anti-inflammatory M2 macrophages. Thus, incorporation of cytokines such as IL-4 or IL-13 into bioscaffolds may be an important consideration if anti-inflammatory or tissue repair macrophage phenotypes are desired. Bonito et al. [48] showed that the functionalisation of synthetic 3D scaffolds with IL-4 resulted in a dose-dependent increase in human monocyte-derived macrophage TGF-β secretion, indicating the activation of anti-inflammatory phenotypes. Such developments hold great promise for regenerative medicine whereby the implantation of such pro-wound healing macrophage-laden scaffolds would have a greater likelihood of favourable host interactions.

TRM homeostatic functions and self-renewal are thought to be continually maintained by tissue-specific exogenous factors such as cytokines [49]. For example, microglia are shown to require neuron-produced IL-34 for their proper development and maintenance [50], while other myeloid cells such as monocyte-derived macrophages are not sensitive to this cytokine [51]. This would suggest that IL-34 is a cytokine that should be incorporated into 3D cultures for the propagation of microglia. In the bone, receptor activator of NF-κB (RANK) and RANK ligand (RANKL) signalling induces downstream activation of nuclear factor of activated T cells 1 (NFATc1), which collaborates with other transcription factors such as microphthalmia-associated transcription factor (MITF) to regulate osteoclast-specific genes and maintain osteoclast identity [52]. Osteoclast culture in 2D is well established and as a minimum requires RANKL together with CSF1 [53]; it would therefore follow that these molecules should be considered for the 3D culture of osteoclasts. While microglia and osteoclasts have reasonably well-defined *in vitro* cytokine requirements, it is less clear for other TRMs. In a recent study, the renewal of local liver TRM (Kupffer cell) populations post partial-hepatectomy required IL-6 [54]. Kupffer cells are also shown to need bone morphogenetic protein (BMP) 9 and BMP10 cytokine signalling for identity and self-renewal via activin receptor-like kinase 1 (ALK1) [55]. In contrast, lung TRMs (alveolar macrophages) were shown to require CSF2 (also known as granulocyte-macrophage colony stimulating factor) *in vivo* to both acquire their identity in the developing tissue and to maintain their viability throughout adulthood [56]. In support of this notion, the supplementation of CSF2 into *in vitro* 2D cultures resulted in the long-term expansion of cells that largely maintain alveolar macrophage identity [57]. Overall, these studies highlight the importance and tissue-specificity of cytokines required for the maintenance and propagation of TRMs *in vivo*. However, for certain TRMs evidence is limited as to which cytokines are required *in vitro* and so careful optimisation may be is necessary before their inclusion within 3D cultures.

### Interactions with other cells

During wound healing, macrophages have a well-established role in communicating with neighbouring fibroblasts to promote tissue remodelling [58–61]. Reciprocating the relationship, fibroblasts also secrete factors including CSF1 to sustain tissue macrophage populations [62]. However, an elegant study by Zhou et al. [63] showed that fibroblast production of CSF1 is not simply a secreted factor recognised by macrophages, but instead promotes the physical cell:cell interaction of macrophages with fibroblasts. They subsequently proposed that this close spatial proximity is critical for tissues to regulate overall population numbers of both macrophages and fibroblasts [63], meaning that macrophages are sensitive to the presence of fibroblasts within tissues, and vice versa. In a recent follow-up study, this same group elucidated that fibroblast production of CSF1 is dependent on their sensing of the 3D tissue environment and available space [64]. Such findings could be further interrogated and modelled within 3D culture systems, for example, by tuning scaffold porosity and examining its effect on fibroblast production of CSF1 and subsequent macrophage proliferation.

### Tissue extracellular matrix proteins and their sensing by macrophages

In addition to immunogenic cues, interactions with the tissue extracellular matrix (ECM) have been shown to influence macrophage activation and function. The ECM is composed of a range of different proteins including fibrous protein assemblies (e.g. collagens, elastins, fibronectins, laminins) that provide structural architecture for cellular adhesion, influence cellular morphology, migration and signal to cells; and proteoglycans and glycosaminoglycans, which form a gelatinous environment that buffers and hydrates the tissue [65]. In addition, the composition of the ECM may directly modulate macrophage phenotypes [66]. Here, bone marrow-derived macrophages (BMDM) cultured with decellularized small intestine ECM adopted genetic signatures like anti-inflammatory ‘M2' macrophages, while culture with decellularized urinary bladder ECM promoted transcriptomes closer to pro-inflammatory ‘M1' stimulated macrophages [66]. In another study, macrophages exposed to ECM proteins from the small intestine, urinary bladder, brain and colon were found to adopt anti-inflammatory M2 phenotypes, whereas dermal ECM promoted M1 [67]. In these two studies, distinct macrophage phenotypes are induced upon exposure to different decellularized tissues, however, due to the crude nature of these supplements it remains unclear whether these results are due to the ECM composition of tissues or other factors. In a more direct study, macrophage culture on fibronectin aggregates with IFNγ stimulation displayed enhanced phagocytosis and nitric oxide secretion compared with those stimulated with IFNγ alone [68]. In another example, the molecular weight (MW) of the extracellular matrix glycosaminoglycan hyaluronic acid influenced macrophage polarisation where low MW hyaluronic acid promoted M1 activation, while high MW hyaluronic acid induced anti-inflammatory M2 activation [69]. Taken together, these studies demonstrate that ECM materials can influence macrophage functions in synergy with immunogenic stimuli or alone and thereby the selection of ECM protein can significantly sway the desired outcome. In addition, a shortcoming with these various studies is that they differ greatly in the concentration of ECM proteins used making their replication difficult. Therefore, the incorporation of ECM materials into 3D scaffolds must be carefully considered depending on the tissue of interest, and active concentrations optimised to avoid unwanted macrophage activation phenotypes.

**Figure 2.**
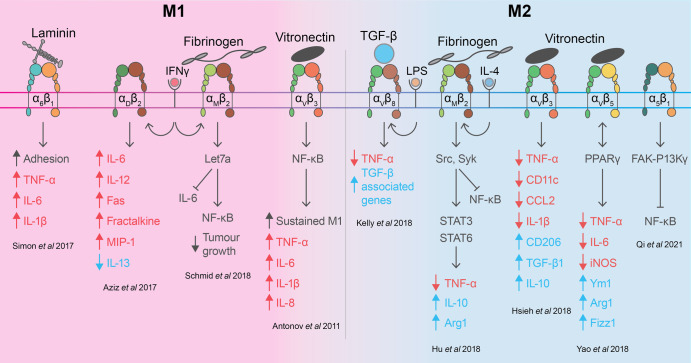
Macrophage activation markers regulated by integrin expression and ligation. Macrophages interact with extracellular matrix proteins through surface integrins, transmembrane proteins consisting of an alpha (α) and beta (β) subunit. Upon activation by extracellular signals such as interferon gamma (IFNγ) and lipopolysaccharide (LPS), macrophages adopt ‘M1' phenotypes, characterised by up-regulated pro-inflammatory cytokine production such as interleukin (IL)-6 and tumour necrosis factor alpha (TNFα). M1 macrophages also secrete chemokines including fractalkine and macrophage inflammatory protein (MIP)-1 to further recruit immune cells. Integrins α_D_β_2_, α_M_β_2_ and α_V_β_3_ up-regulate expression of these classical M1 markers via the nuclear factor kappa-light-chain-enhancer of activated B cells (NF-κB) pathway, which can result in supressed tumour growth. Alternatively, macrophages activated by IL-4 adopt an ‘M2' phenotype, characterised by up-regulated anti-inflammatory cytokine production, including transforming growth factor (TGF)-β and IL-10, and M2 markers arginase 1 (Arg1), Ym1 and Fizz1. TGF-β receptor α_V_β_8_ promotes M2 phenotypes by supressing pro-inflammatory activation by LPS, whilst vitronectin receptor α_V_β_5_ enhances M2 activation through peroxisome proliferator-activated receptor gamma (PPARγ).

Considering reports that ECM composition influences macrophage function, perhaps unsurprisingly it has also been shown that integrins are also important in steering macrophage activation (Figure 2). Integrins are surface receptors expressed by macrophages and many other cells which bind ECM proteins [70]. Integrins are composed of two subunits, where one of 18 different alpha (α) subunits is combined with eight different beta (β) subunits, forming a multitude of functionally distinct heterodimers that form specific receptors for ECM proteins. For example, α_1_β_1_ integrin interacts with specific peptide sequences on collagen I, while α_v_β_3_ binds different sequences on vitronectin. Therefore, how macrophages respond to specific ECM proteins within tissue environments is highly dependent on integrin expression.

There have been numerous reports identifying various integrins to mediate macrophage activation, however there is generally a lack of consensus in the field as to which integrins are important, and the molecular mechanism by which they support macrophage phenotypes. For example, macrophages stimulated with LPS and IFNγ up-regulate integrin α_M_β_2_ (also known as CD11b/CD18), which binds fibrinogen and intercellular adhesion molecule (ICAM)-1; resulting in enhanced macrophage anti-tumour activity via NF-κB signalling [71]. In addition, many integrins bind multiple ECM ligands making understanding their precise role in macrophage activation more difficult. This includes α_D_β_2_ (also known as CD11d/CD18; ICAM-3, VCAM-1 receptor) and α_V_β_3_ (vitronectin, fibronectin receptor), both of which have been shown to positively regulate macrophage inflammatory responses including M1 cytokine expression [72,73]. α_D_β_2_ is substantially up-regulated on macrophages during inflammation and retained at sites of inflammation, promoting chronic diseases such as atherosclerosis [73]. Moreover, multiple integrins can bind to a single ECM protein. For example, while α_V_β_3_ and α_V_β_5_ both bind the ECM protein vitronectin, α_V_β_3_ is associated with pro-inflammatory macrophage activation, and α_V_β_5_ is up-regulated in anti-inflammatory M2 macrophage activation [74]. This could be due to different peptide sequences on the same ECM protein detected by specific integrins [75]. In addition to being ECM receptors, integrins also have the capacity to detect other ligands, including transforming growth factor beta (TGF-β). Kelly et al. [76] showed that TGF-β binding to α_v_β_8_ reduced TNF-α production in response to LPS, suggesting this ligation supresses pro-inflammatory M1 activation. Overall, these contrasting studies highlight the confusion in the field as to which integrins are important for macrophage phenotypes. Elucidation of which macrophage integrins must be ligated for specific functions is a critical consideration in the design of 3D models, as unligated integrins can cause cellular apoptosis and death.

As well as integrins, macrophages also express tetraspanins on their cell surface, a family of proteins which interact with and functionally regulate integrins and a range of other signalling molecules [77,78]. Studies have shown that tetraspanins can also regulate macrophage activation. For example, tetraspanin CD9 was shown to supress pro-inflammatory macrophage activation by reducing TNFα and matrix metalloproteinase production during HIV infection [79]. Additionally, tetraspanin CD81^−/−^ macrophage-like cell lines exhibited significantly higher proliferation rates [80]. Interestingly, knockouts of both CD9 and CD81 simultaneously resulted in the formation of multinucleated giant cells in the lung, showing that these two tetraspanins work together to prevent phagocyte fusion [81]. Our own research has shown that CD82^−/−^ macrophages have decreased anti-inflammatory M2 phenotypes [82]. In this case, we observed a decreased expression of macrophage integrin α5, suggesting that CD82 regulates the expression of this integrin in M2 activated macrophages. This further highlights the importance of sensing and binding to ECM proteins for the activation of macrophage phenotypes. Elucidation of the plasma membrane proteome of monocyte-derived macrophages and TRMs from different tissues, and during various challenges, is necessary to uncover the molecular mechanism by which ECM interactions support macrophage function.

### Mechanical properties

Apart from the composition of the tissue microenvironment, mechanical properties such as stiffness (measured in Pascal, Pa) also affect macrophage biology. In humans, residing TRMs or infiltrating monocyte-derived macrophages will experience a variety of stiffnesses depending on the organ: liver ∼1 kPa, lung ∼5 kPa, muscle ∼20 kPa, while uncalcified bone is >100 kPa [83]. The mechanical properties of these tissues are influenced by ECM composition and their microstructure [84]. The stiffness of culture conditions has recently been shown to influence macrophage phenotype. In a study by Gruber et al. BMDMs cultured in 2D on soft substrates (1 kPa) displayed rounder and smaller morphologies compared with those grown on stiffer substrates (150 kPa). Furthermore, BMDMs stimulated with toll-like receptors TLR4 and TLR9 agonists on softer cultures secreted higher levels of the pro-inflammatory cytokine TNFα, indicating that the elasticity of the culture substrate modulated macrophage sensing of PAMPs [85]. In contrast, plastic 2D dishes routinely used to culture cells *in vitro* are >1000 kPa. More recently, Meli et al. [86] showed that the pro-inflammatory activation of human monocyte-derived macrophages was reduced upon culture on soft fibrin substrates. In addition, they identified that the activity of the mechanosensing transcription factor Yes-associated protein (YAP) correlated with increasing substrate stiffness and, subsequently, pro-inflammatory TNF-α production [86]. Mechanosensing and mechanotransduction explains why cells (including macrophages) have altered phenotypes when cultured in substrates of different stiffnesses. Generally, it is considered that the connection cell cytoskeletons to the ECM via integrins results in the formation of focal adhesions [87]. This subsequently regulates ‘outside-in' and ‘inside-out’ integrin signalling, which ultimately activate transcription factors such as YAP that mediate the cells response to the environment (reviewed in [88,89]). Altogether, further identification of molecules and mechanisms that are responsible for sensing the physical environment of tissues will be essential in our understanding of how this factor supports macrophage activation.

### Tissue geometry

During diseases such as cancer and tissue fibrosis, the shape or architecture of tissues can change due to increases in collagen production and ECM crosslinking [90,91]. Considering that macrophage phenotype plays a role in both these conditions, the effect of tissue geometry on macrophage activation has been explored by several studies. a role in driving both these environments are known to promote anti-inflammatory, pro-fibrotic macrophage phenotypes, perhaps it is unsurprising that changes to tissue geometry influence macrophage biology [92,93]. In a study by Wang et al. [93] after transplanting shrink-film wrinkles into the subcutaneous layer of mice, collagen deposition decreased at those sites while arginase-1 expression was increased, suggesting optimal macrophage conditions to avoid foreign body responses in future transplants. Elsewhere, forcing elongated morphologies via ECM ‘stamps' caused macrophages to acquire M2 phenotypes without the aid of cytokine supplementation [92]. These two studies highlight the importance of geometry in steering macrophage phenotypes, even in the absence of immunogenic cues. 3D bioprinting is one method which could be used to further interrogate the role of geometry in macrophage activation.

## Application of 3D models to understand macrophage biology

### Tumour biology

3D cell culture has matured largely in the cancer research field as the tumour microenvironment is well recognised to be crucial for tumour development and immunity [94,95]. For example, aggregates of tumour cells (also known as spheroids) are more frequently being employed in high-throughput drug screens to identify new chemotherapeutics [98]. Macrophages are important immune components of the tumour microenvironment where their phenotype is a determinant for tumour growth or removal [99]. To recreate this interplay *in vitro*, Linde et al. [100] cultured human squamous cell carcinoma (SCC) cells together with macrophages and fibroblasts in 3D collagen I hydrogels. Unactivated macrophages suspended within these 3D SCC co-culture scaffolds were founds to spontaneously adopt a M2 phenotype, recapitulating how cancer cells program favourable pro-tumour macrophage phenotypes *in vivo* [100]. In another study, M2 macrophage cytokine SPP1 accelerated the growth of prostatic intraepithelial neoplasia (PIN) when co-cultured on 3D Matrigel scaffolds [96]. Elsewhere, a similar prostate cancer model revealed macrophage cytokines C5a, CXCL1 and CCL2 responsible for PIN cell proliferation through ligand–receptor interactions [97]. Together, these preliminary studies provide evidence for the development of 3D models that effectively mimic complex tumour–macrophage interactions observed *in vivo*. Such models would be important in future studies for the screening of new chemotherapies and immunotherapies.

### Infection and granuloma formation

Macrophages are often the host cell of invading pathogens since they are the first to engulf the foreign entity. Another application of 3D macrophage models could be the development host-directed therapies for infectious diseases – thereby bypassing mechanisms of pathogen resistance and boosting macrophage microbicidal responses [101]. This may be particularly important in infections with intracellular pathogens such as *Mycobacterium tuberculosis (Mtb)* and *Leishmania spp.* which induce granuloma formation. These large aggregates of immune cells (including macrophages) aim to limit the spread of persistent infections, but in doing so, can result in long-lasting latent infections that are increasingly harder to treat [102]. To generate a 3D human tuberculosis granuloma model, Tezera et al. [103] used microsphere technology where spheroids containing *Mycobacterium tuberculosis* (*Mtb*) and primary human blood mononuclear cells (PBMCs) where formed within a collagen-alginate matrix. These spheroids exhibited *in vivo* granuloma-like characteristics, including prolonged culture of human cells and increased cytokine production. Elsewhere, *Mtb*-infected human PBMCs seeded in collagen matrices formed microgranulomas comprising of macrophages and T cells that exhibited increased inflammatory cytokine production and accumulation of lipid bodies, typical of latent *Mtb* infection [104]. More recently, spheroids generated with THP-1 monocyte/macrophage cells have been used to study *Mtb* coinfection with other diseases such as HIV, highlighting the capacity of 3D cell culture to understanding complex biological processes [105]. Given that it is becoming increasingly recognised that no animal model can fully reproduce human *Mtb* infection *in vivo*, new 3D models of *Mtb* provide promising alternatives in which human infection can be accurately recapitulated in a controlled *in vitro* environment [106,107]. In all, the application of 3D systems such as these permit new dissection of host–pathogen interactions during infectious diseases. One such example is COVID-19, where severity of infection is correlated to the function of lung TRMs known as alveolar macrophages. Alveolar macrophage responses during SARS-CoV-2 infection is proposed to result in the overproduction of inflammatory cytokines or so-called ‘cytokine storm' and increase the severity COVID-19 [108]. Furthermore, it has been shown that alveolar macrophages are less effective at combating SARS-CoV-2 infection post-inflammation, leading to long-term susceptibility to disease [109]. Lung tissue contains a high amount of elastin, collagen and glycosaminoglycans to give its characteristic elasticity [110,111]. Multiple methods have been developed to decellularize lung tissue to analyse the effect of ECM composition, however there is a lack of studies exploring the influence of this environment on lung TRM biology [111,112]. Considering the significant role alveolar macrophages play in clearing respiratory diseases and the uniqueness of the lung extracellular environment, 3D models may be used to further improve our knowledge of diseases like COVID-19, thereby improving therapeutic discovery.

## Perspectives

- Macrophages are cells which reside or infiltrate all mammalian tissues and are critical for maintaining the homeostasis of their microenvironment, thereby making them prime candidates for 3D culture.
- The development of 3D models for macrophage culture would aid our understanding of their function in the context of many diseases (e.g. cancer, infection) and during homeostasis.
- With the latter, there is still a great deal we don't know about the tissue-specific requirements for TRM function and renewal, and whether they can be recompensed by monocyte-derived counterparts. Careful recapitulation of specific environmental cues such as ECM protein composition and elasticity in 3D models would enhance our understanding of the biology of these important cells.

## Open Access

Open access for this article was enabled by the participation of La Trobe University in an all-inclusive *Read & Publish* agreement with Portland Press and the Biochemical Society under a transformative agreement with CAUL.

## Abbreviations

CSF1: colony-stimulating factor 1
DAMPs: damage associated molecular patterns
ECM: extracellular matrix
ICAM: intercellular adhesion molecule
IL: interleukin
LPS: lipopolysaccharides
MW: molecular weight
NO: nitric oxide
PAMPs: pathogen associated molecular patterns
PBMCs: primary human blood mononuclear cells
PEG: polyethylene glycol
PIN: prostatic intraepithelial neoplasia
PRRs: pattern recognition receptors
RANK: receptor activator of NF-Κb
SCC: squamous cell carcinoma
TGF: transforming growth factor
TLR: toll-like receptor
TNF: tumour necrosis factor
TRM: tissue resident macrophage
YAP: Yes-associated protein
